# Apple pomace improves the quality of pig manure aerobic compost by reducing emissions of NH_3_ and N_2_O

**DOI:** 10.1038/s41598-017-00987-y

**Published:** 2017-04-13

**Authors:** Hui Mao, Teng Zhang, Ronghua Li, Bingnian Zhai, Zhaohui Wang, Quan Wang, Zengqiang Zhang

**Affiliations:** 1 College of Resources and Environment, Northwest A& F University, Yangling, Shaanxi Province 712100 China; 2 Key Laboratory of Plant Nutrition and the Agri-environment in Northwest China, Ministry of Agriculture, Yangling, 712100 China

## Abstract

In this study, the effects of apple pomace (AP) addition (0%, 5%, 10%, and 20% on a dry weight basis, named as control, AP1, AP2, and AP3) and citric acid (CA) addition on nitrogen conservation were investigated during aerobic composting of pig manure. Gaseous emissions of NH_3_ and N_2_O were inhibited by AP and CA addition, with AP’s effect greater. The inhibition improved with increasing AP addition. The AP3 treatment was the most effective on NH_3_ adsorption and transformation to ${{\bf{NH}}}_{{\bf{4}}}^{{\boldsymbol{+}}}$-N, improved with subsequent transformation to ${{\bf{NO}}}_{{\bf{3}}}^{{\boldsymbol{-}}}$-N, and inhibition of N_2_O and ${{\bf{NO}}}_{{\bf{2}}}^{{\boldsymbol{-}}}$ production. Compared with control, AP3 showed the highest inhibition of accumulated NH_3_ and N_2_O emission, by 57% and 24%, respectively, and with a 19% increase of total Kjeldahl nitrogen in the compost. The further pot experiment proved the application of the AP amendment compost could improve the yield and trace element nutrient accumulation in Chinese cabbage when planted in a typical Zn-deficient soil. This study illustrates that AP application benefits both compost nitrogen conservation and fertilizer quality.

## Introduction

China is the biggest pork producing country in the world and approximately 715 million tons of pig manure (PM) were produced in 2013^1^. Overproduction of PM has led to serious environmental problems. Reducing the amount of PM and control the associated pollution has been regarded as one of the limiting factors for further development of the pig enterprise^2^. Composting is a traditional, effective method, which can not only reduce the amount of organic waste, but also transform animal manure and organic waste into an environmentally friendly organic fertilizer^3, 4^.

In the process of composting, feed stock materials suffer degradation during the thermophilic stage, which leads to the loss of N (mainly in form of NH_3_ volatilization), which lowers compost quality and increases atmospheric pollution^5^. In order to reduce the N loss during composting, various methods have been tried in recent years such as altering process conditions or the addition of different bulking agents and additives. For example, Shi *et al*. reported that increasing moisture and turning the pile can reduce N loss under a long maturity period with cow manure composting^6^. Ventilation control was an effective way to reduce ${{\rm{NO}}}_{3}^{-}$-N loss at the later stage of compost making^7^. Other methods include using mineral or biological amendments to absorb ammonia^8^. For example, the microbial inoculant additive, urease inhibitors can help NH_3_ emission reduction^9^. Some adsorbents including zeolite, bentonite, peat, fly ash, biochar, etc.^10–12^ also could significantly reduce compost NH_3_ emission due to their extensive porosity and large surface area which can provide large NH_3_ adsorption capacity. Besides these, some chemical additives such as Ca_3_(PO_4_)_2_, MgCl_2_, FeCl_3_, Al_2_(SO_4_)_3_, HNO_3_, and CaCl_2_, etc.^13^ could be employed in composting for NH_3_ emission reduction through their chemical reactions with NH_3_. Although these commercial additives (mineral and biological) are effective in improving compost quality by inhibiting N loss, their usage is still restricted due to high cost and risk of excessive accompanying salt ions^4^. In view of economical waste disposal, more research is needed to enhance capacity of N conservation in compost, reduce composting cost and improve environmental friendliness^14^.

Recent studies showed that reducing the initial pH of the compost mixture by adding olive pomace can conserve N^15^. In addition, this method could lead to maximizing utilization of local resources. China is the largest apple producer in the world and total production reached 31.7 million tons in 2013^16^, and Shaanxi province accounted for more than 1/3 of the country’s total apple output^1^. As a by-product of the cider-processing industry, more than 70,000 tons of apple pomace (AP) are generated each year in Shaanxi province, which need to be dealt with^17^. Recently, some reports pointed out that AP may be a suitable material to aid composting. Kopčić *et al*., for example, studied the temperature variation of laboratory-scale in-vessel co-composting of tobacco and apple waste^18^. Hanc and Chadimova vermicomposted AP waste with wheat straw and found that the addition of straw to AP did not enhance earthworm biomass^19^, but did increase the available content of nutrients (N, P, K, Mg etc.) during composting. Jiang *et al*. proved that AP can effectively reduce the NH_3_ release during the pig manure composting^17^. However, emission of N_2_O during composting reduced N conservation and contributed to greenhouse gas (GHG) emission^20^. There has been little research on reducing N_2_O during composting AP with PM which has limited the application of AP in composting.

Hence, the aims of this study were to investigate the effect of AP addition with different amounts on the gaseous emissions of NH_3_ and N_2_O during the compost N conservation process. In the composting, treatments with different amounts of AP addition were evaluated by aerobic composting of the mixture of PM and wheat straw (WS) in compost reactors. Concurrent treatment with citric acid (CA) was included in order to compare the effects of AP with an organic acid in composting.

## Results and Discussion

### Changes in compost temperature, pH, EC and germination index (GI)

**Figure 1:**
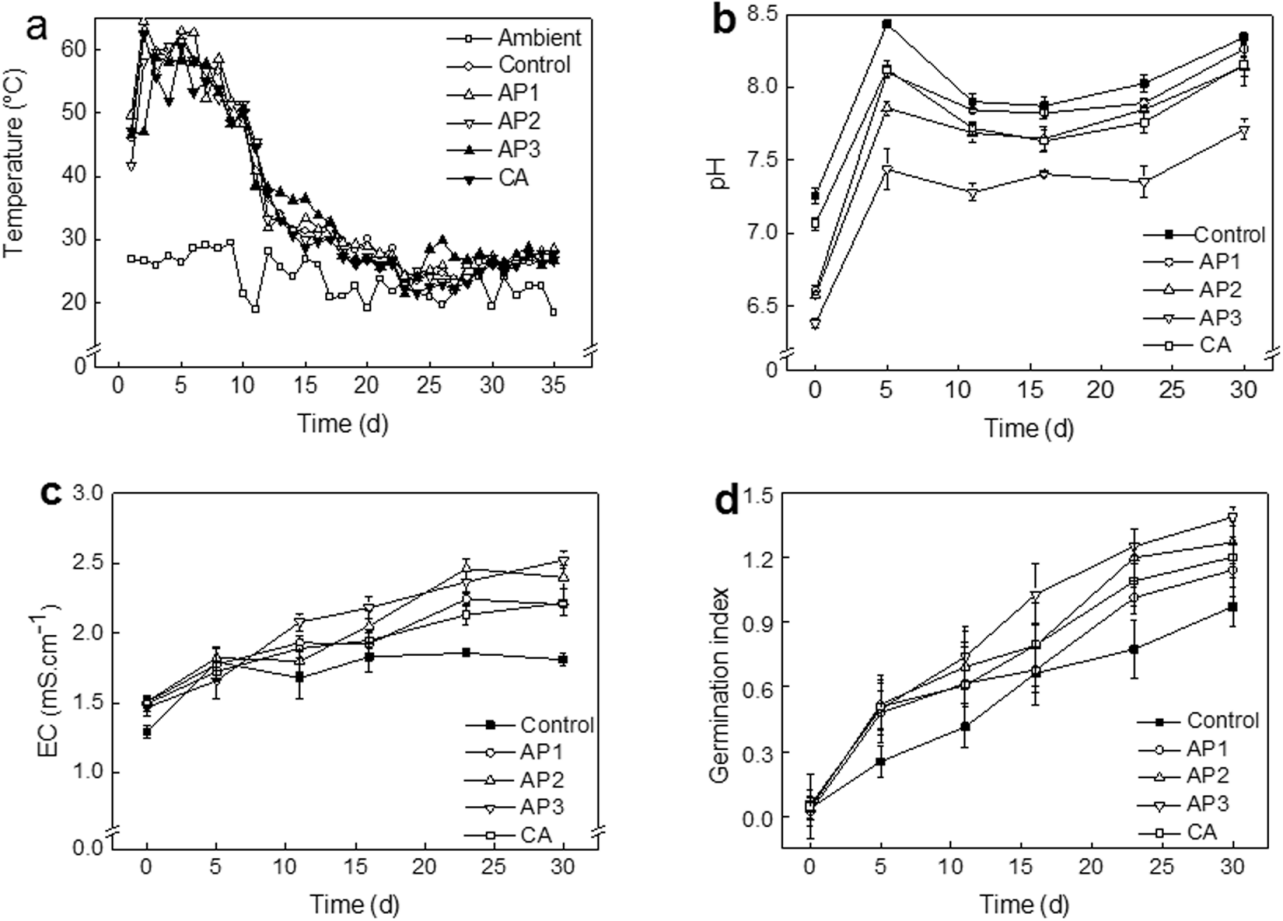
Profiles of temperature (**a**), pH (**b**), EC (**c**), and germination index (**d**) during the composting process.

The changes of temperature observed in the five treatments are shown in Fig. 1a. The ambient temperature was maintained from 20 °C to 30 °C during the process. The highest temperatures were obtained after 2 days with 62.6, 62.9, 61.4, 58.8, and 60.6 °C in control, and treatments of AP1, AP2, AP3, and CA, respectively. The thermophilic phase was maintained about one week for the feedstock composition, which was necessary for obtaining a successful product^21^. There were no differences with time for all treatments to reach the thermophilic phase. The temperature decreased rapidly after 10 days, indicating the end of the thermophilic phase, and then gradually decreased to about 30 °C. The additive amended treatments did not show any stimulatory or inhibitory effects on the temperature profile, which was similar to that reported by Li *et al*.^4^.

The pH of the compost mixture appeared to increase sharply in the first 5 days and then decrease slightly to gradually reach pH 7.7 to 8.3 in the later process (Fig. 1b). In the first phase, the mineralization of organic matter generally leads to the release of ammonium and volatile ammonia, which increases pH level^22^. With the composting prolonged, pH decreased due to the formation of low molecular weight fatty acids and CO_2_ during organic matter (OM) degradation, and the accumulation of organic acid with the addition of AP. At the end of the process, pH increased with the decomposition of organic acid. For AP additive treatments, AP3 was significantly lower than other treatments at the later composting process, due to more organic acid produced with more AP.

The change of electrical conductivity (EC) during the composting process revealed the amount of soluble salt contents in the compost (Fig. 1c). EC value increased in all treatments with composting time. After composting, the additive treatments had significantly higher EC than control, which reached 2.20, 2.52, 2.58, and 2.22 mS cm^−1^ for AP1, AP2, AP3, and CA treatments, respectively, compared with the relative lower EC of 1.81 mS cm^−1^ for the control. In the end, EC increased by 40% for control, while 46%, 67%, 72%, and 48% for AP1, AP2, AP3, and CA treatments, respectively. Increases in EC were caused by organic matter mineralization that can increase concentration of soluble salts^23^. Compared to the different additive treatments, AP3 obtained the highest increasing rate, which can be explained by more organic acid being produced with more AP addition, which can lead to an increase of soluble salts concentration in the compost^17^.

Figure 1d clearly shows a tendency of gradual decrease in compost phytotoxicity. Compared with the control, addition of AP and CA improved germination index (GI) variation, and at the end of composting the GI value reached 0.97, 1.14, 1.27, 1.38, and 1.20 for control, AP1, AP2, AP3, and CA, respectively. Generally, acceptable GI value of mature compost was above 0.5^24^. In the study, GI of the final compost were higher than 0.9, indicating the compost in all the treatments was mature. The GI values of the compost with AP and CA additive were higher than that of compost in control, which illustrated that AP and CA amendment can help compost material detoxification.

### Variation of NH_3_ and N_2_O emission during composting

**Figure 2:**
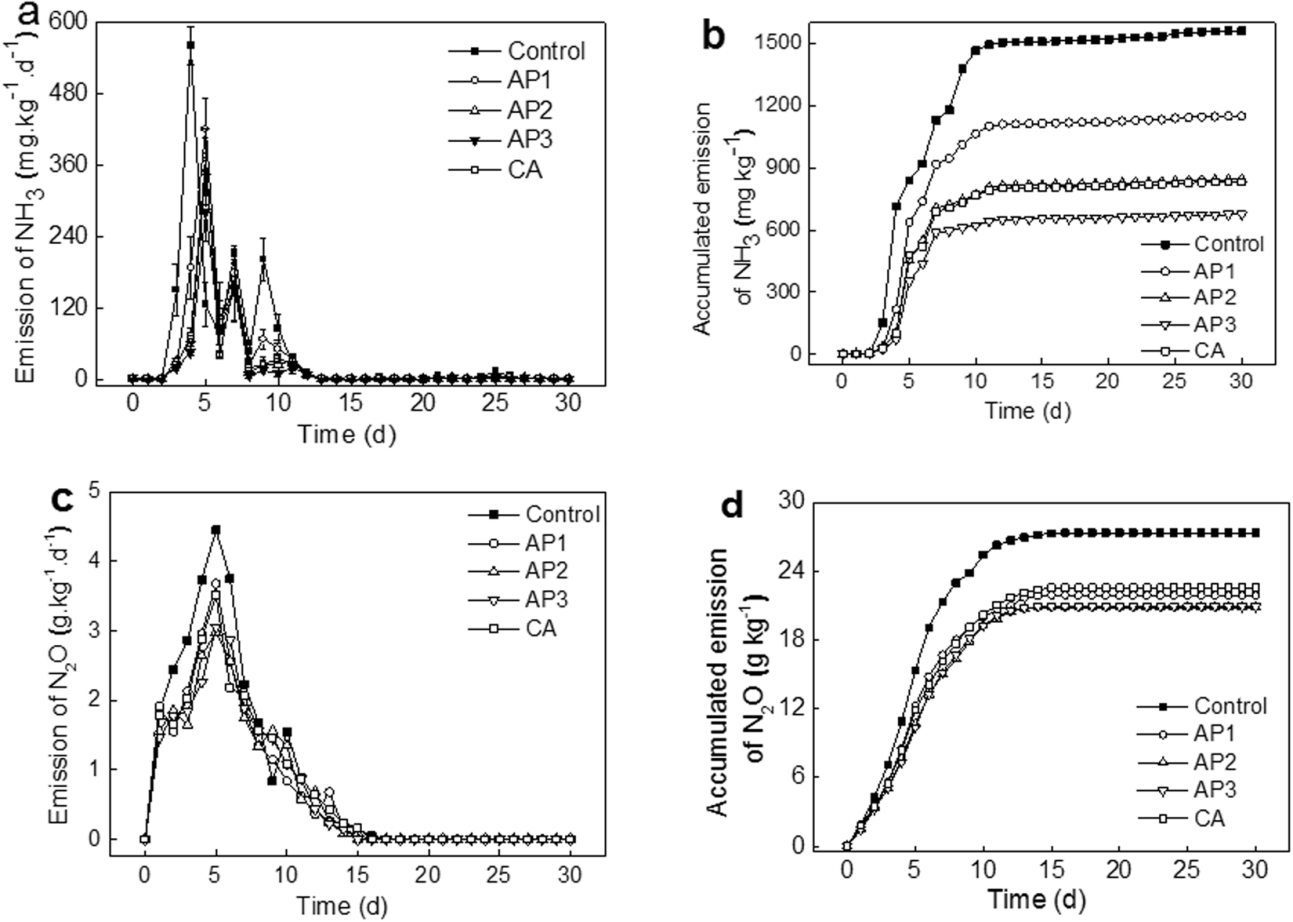
Effects of additives on NH_3_ emission (**a**) and accumulated amount (**b**), N_2_O emission (**c**) and accumulated amount (**d**) during composting.

The NH_3_ and N_2_O emissions were important in this study both for the effect of N transformation and global warming influence. As shown in Fig. 2a, ammonia concentrations peaked after 5 d during the composting. Compared with control, NH_3_ concentrations were significantly reduced in the additives treatments, especially in the AP3. The emissions of NH_3_ from animal manure were influenced by pH and the ${{\rm{NH}}}_{4}^{+}$-NH_3_ transformation equilibrium^12^. The amendments, especially AP and CA used in this study, had a pH under 7, which tended to prevent NH_3_ losses^21^. However, the losses of NH_3_ in the AP3 treatment was lower, which might be caused by the NH_3_ adsorption potentials at lower pH since the amount of AP was higher than other treatments and the compost pH at the start was lower. This phenomenon was in accordance with the findings in previous reports during composting of olive pomace with animal waste^15^. Beside this, the AP additive with its smaller particle size compared with wheat straw and pig manure, can lead to high bulk density of mixed initial compost material which can reduce NH_3_ emission, as reported, by 30–70%^10^. The accumulated emission of NH_3_ was calculated to clarify the effect of treatments (Fig. 2b). There existed similar curve with all treatments that increased rapidly in the first 7 days and then reached a platform. The total emission of NH_3_ of all the additive treatments of AP and CA showed significant inhibition with control. The inhibited rates with control were 26%, 46%, 57%, and 47%, for AP1, AP2, AP3, and CA treatments, respectively. The results confirmed that AP and CA additive can help to reduce NH_3_ emission during composting, which probably indicated there was more organic acid in the initial materials or more was produced during the composting process.

The N_2_O emission profile in the composting process was studied by many researchers for its important effects both in N conservation during the process and GHGs emission for its global warming influence^25^. As reported by former studies, N_2_O can be produced under both aerobic and anaerobic conditions^26^. During denitrification, N_2_O can be synthesized where there is a lack of O_2_ and/or a nitrate (or nitrite) accumulation^27^, while during nitrification, N_2_O is produced in the presence of O_2_ and/or low availability of degradable carbohydrates. In this study, N_2_O emission increased rapidly in the first phase with all treatments and peaked in the fifth day, reached with 4.45, 3.68, 2.98, 3.05, and 3.52 g kg^−1^ d^−1^ for the treatments of control, AP1, AP2, AP3, and CA, respectively (Fig. 2c), and decreased gradually after the peak time. All the N_2_O emission profile approached zero after 15^th^ days in composting. The accumulated emission of N_2_O was shown clearly with all treatments (Fig. 2d). The additive treatments inhibited the N_2_O accumulation compared with control, by 20%, 24%, 24%, and 18% for AP1, AP2, AP3, and CA, respectively. It should be pointed out that the mechanism and tendency of N_2_O emission during composting is still disputed, according to earlier reports. El Kader *et al*. reported that a high concentration of N_2_O was found at the beginning of composting with farm manure^28^. Our study also showed the N_2_O emission increased rapidly in the first phase with all treatments, and the N_2_O emission could be decreased with AP addition. These results were in accordance with the finding of Awasthi^20^, who reported the high concentration of N_2_O was found at the beginning of composting with sewage sludge, while some additives such as 30% zeolite and 1% lime could reduce greatly the N_2_O emission during the first composting stage. However, N_2_O was mostly reported previously to be emitted during the thermophilic phase, due to the activity of nitrifiers was inhibited by high temperature^29, 30^. These results suggest that the N_2_O emission in composting is a complicated system which could be influenced by many factors.

### Variation of TKN, ${{\bf{NH}}}_{{\bf{4}}}^{{\boldsymbol{+}}}$-N, ${{\bf{NO}}}_{{\bf{2}}}^{{\boldsymbol{-}}}$-N, and ${{\bf{NO}}}_{{\bf{3}}}^{{\boldsymbol{-}}}$-N during composting

**Figure 3:**
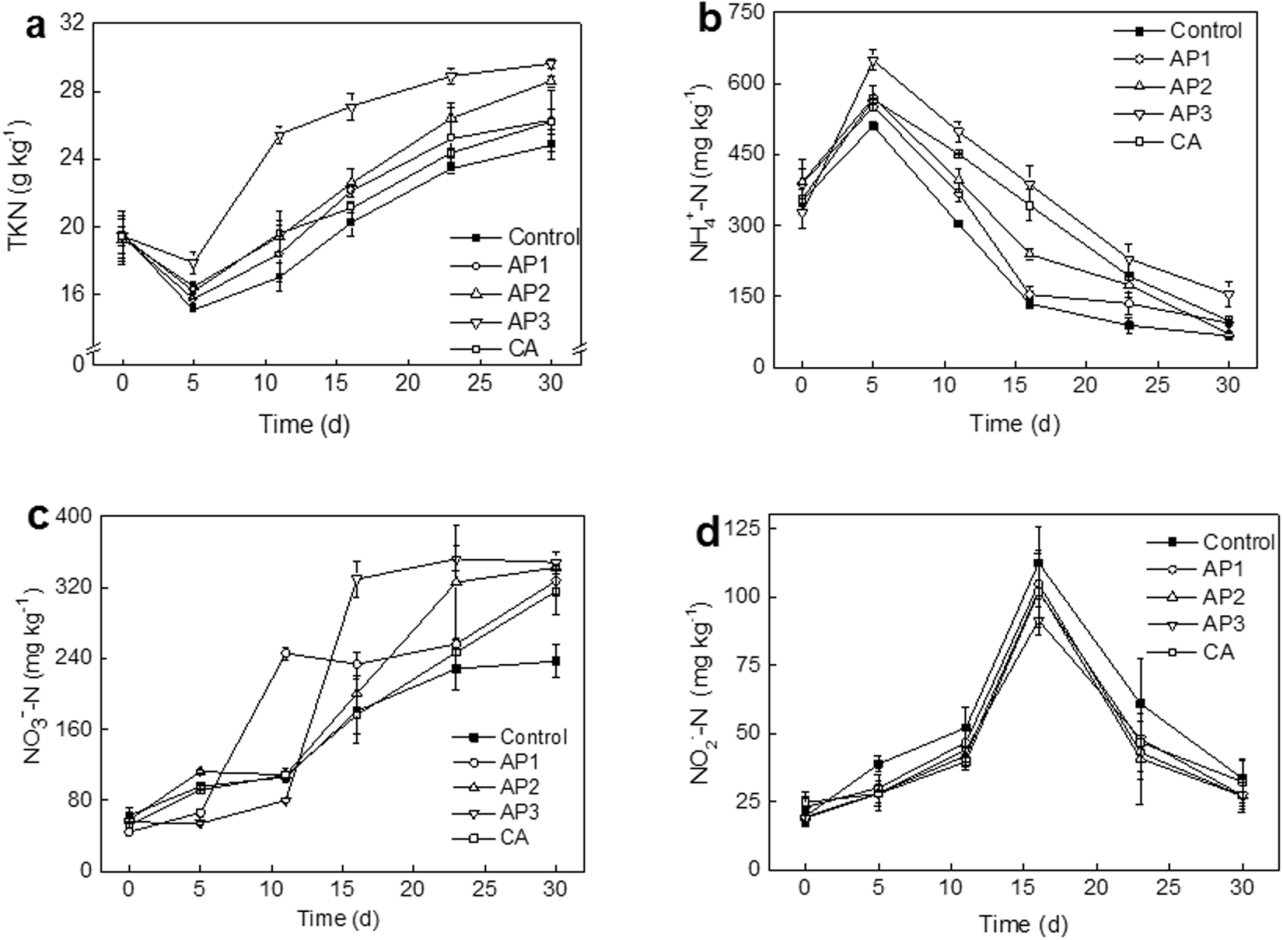
Effects of additives on TKN (**a**), ${{\rm{NH}}}_{4}^{+}$-N (**b**), ${{\rm{NO}}}_{3}^{-}$-N (**c**), and ${{\rm{NO}}}_{2}^{-}$-N (**d**) during composting.

The total Kjeldahl nitrogen (TKN) contents in the composting mixtures with all treatments decreased during the first 5 days and increased afterwards (Fig. 3a). The loss of TKN at the beginning of the composting stage might be due to the loss of ammonia by volatilization with increasing compost temperatures. Compared with the control, the TKN losses of AP and CA adding treatments in the first five days were lower than that in the control, which were 19%, 15%, 8%, and 16% in AP1, AP2, AP3, and CA, respectively, while 22% in control. Similar results were reported by Chen *et al*.^11^, that TKN loss from treatments of 3%, 6%, and 9% bamboo charcoal addition in pig manure composting were reduced by 28%, 61%, and 65%, respectively, compared to the control after composting. The increase of TKN at the later stage in all treatments was possibly because of the continuous degradation of organic compounds^31^, while in this study, with the AP addition, the TKN content in the composting mixtures remained higher than other treatments throughout the entire composting. In the end of composting, the TKN contents were 24.9, 26.3, 28.6, 29.6, and 26.2 g kg^−1^ with the treatments of control, AP1, AP2, AP3, and CA, respectively. Similar results were obtained by other researchers with the compost additives of biochar^12^, bentonite^2^, bamboo charcoal^32^. This might be caused by the fact that the AP could act as an ammonia sorbent which inhibited ammonia emission in the early phases^17^, or the acidic AP could inhibit the ammonia release through the reaction H^+^ + NH_3_ = ${{\rm{NH}}}_{4}^{+}$ ^21^. And this point correlates with ${{\rm{NH}}}_{4}^{+}$-N evolution to some extend during composting, as shown in Fig. 3b.

The ${{\rm{NH}}}_{4}^{+}$-N contents increased during the first 5 d and the peak ${{\rm{NH}}}_{4}^{+}$-N contents were 510.6, 550.4, 569.9, 649.6, and 567.0 mg kg^−1^ in the control, AP1, AP2, AP3, and CA treatments, respectively (Fig. 3b). The higher ${{\rm{NH}}}_{4}^{+}$-N content of the compost occurred during the first 5 d, which is in accord with the traditional composting process. In general, the evolution of N forms shows the mineralization of the organic compounds during the active phase of composting with the formation of ${{\rm{NH}}}_{4}^{+}$-N. This was because the higher pH and temperature during the thermophilic phase resulted in more NH_3_ volatilization release, then NH_3_ was subsequently adsorbed by the wet mixture, and thus formed ${{\rm{NH}}}_{4}^{+}$ ^33^. However, in the AP and CA added treatments, the peak concentration of ${{\rm{NH}}}_{4}^{+}$-N was higher than that in control, which suggests that the acid addition helps the NH_3_ immobilization through reaction NH_3_ + H^+^ = ${{\rm{NH}}}_{4}^{+}$. After 5 d, the ${{\rm{NH}}}_{4}^{+}$-N contents rapidly decreased as composting progressed, and only small amounts of ${{\rm{NH}}}_{4}^{+}$-N existed in the final composts (lower than 200 mg kg^−1^), which proved the maturity and stability of the final composts^21^. The later ${{\rm{NH}}}_{4}^{+}$-N content decrease was relevant with the ${{\rm{NH}}}_{4}^{+}$-N microorganism immobilization and ${{\rm{NO}}}_{3}^{-}$-N formation during the aerobic composting process^34^. Generally, nitrate nitrogen is the final sink of inorganic N in the composting system. The ${{\rm{NO}}}_{3}^{-}$-N contents steady increase (Fig. 3c) was accompanied by the reduction of the ${{\rm{NH}}}_{4}^{+}$-N during the aerobic composting process^12, 21^. The result showed that the addition of AP can improve the N reserve with the form of ${{\rm{NO}}}_{3}^{-}$-N in compost according with the decrease of ammonia volatilization. In composting process, the transformation of ammonia to nitrates was caused by the action of nitrifying bacteria with the optimum nitrification pH around 7–8^35^. Gujer & Jenkins^36^ pointed out that a suitable amount of alkali was needed to oxidize ammonia nitrogen and hence reduction of pH would be expected in the nitrification process. In this study, the compost pH decreased as more of the acidic AP was added. The AP1 might provide a suitable pH condition for nitrification in the beginning 10 days than others, which may lead to a higher ${{\rm{NO}}}_{3}^{-}$-N content (~248 mg kg^−1^ at 10 days) in AP1 than that of other treatments. The result partly agreed with the findings reported by Wong *et al*.^37^, who found in treatment of 15% coal fly ash + lime 0.75% the ${{\rm{NO}}}_{3}^{-}$-N content was higher than that in treatment of control, 1.5% lime, 3% lime, 5% coal fly ash + 2.25% lime, and 10% coal fly ash +1.5% lime, respectively, at the beginning of 10 days composting.

${{\rm{NO}}}_{2}^{-}$-N is an intermediate product of N transformation during the composting process^38^. The ${{\rm{NO}}}_{2}^{-}$-N concentration in all treatments increased steadily during the early stage and then declined (Fig. 3d). The peak value of ${{\rm{NO}}}_{2}^{-}$-N in all treatments was observed on day 16 and decreased with the AP adding amount increase, which implicating AP amendment inhibit the ${{\rm{NO}}}_{2}^{-}$-N formation. Meanwhile, during the composting process, the former increase in ${{\rm{NO}}}_{2}^{-}$-N content in all treatments was attributed to the ${{\rm{NH}}}_{4}^{+}$-N converting to ${{\rm{NO}}}_{2}^{-}$-N during nitrification^39^, while the later decrease was caused by the ${{\rm{NO}}}_{2}^{-}$-N transforming to ${{\rm{NO}}}_{3}^{-}$-N or N_2_O through nitrification/denitrification^20, 40^. The ${{\rm{NO}}}_{3}^{-}$-N concentration significant increase (Fig. 3c) indicated the increase in nitrate formation during the continuous aerobic process^21, 38^. At the end of the composting, the AP amendment treatments exhibited higher contents of ${{\rm{NO}}}_{3}^{-}$-N than the control, and the final ${{\rm{NO}}}_{3}^{-}$-N were 236.86, 327.71, 342.53, 348.38, and 315.90 g kg^−1^, for the control, AP1, AP2, AP3, and CA treatments, respectively. The similar trend of nitrate was observed by other researchers during composting organic waste with bentonite^2^, lime^20^ and biochar^11^.

### Effect of the mature compost on Chinese cabbage growth

The effect of AP amended composts on Chinese cabbage growth in pot experiments is given in Table 1. The highest SPAD content in leaves was found with AP3-treated compost, which was 25% and 62% higher than that obtained with control-treated compost and blank treatment, respectively. Compared with the blank treatment, yields of Chinese cabbage treated with composts increased significantly, and the peak weights of biomass were obtained with the AP3-treated compost, and the average weight of AP-treated compost was 26% and 248% higher than that obtained with control-treated compost and blank treatment, respectively. The differences in yields of Chinese cabbage biomass indicated that AP-amended compost effectively promoted Chinese cabbage growth. Li *et al*. also found that ryegrass (*Lolium perenne* L.) yields were significantly improved with 17.1–52.5% higher by the addition of charred biomass (CB) treated compost^12^. Similar results reported by Hua *et al*. that the biomass of fescue grown in substrate with 40% sludge compost (SC) increased 27% in a red soil and 44% in a yellow loamy soil compared to the control, while the biomass of ryegrass grown with 60% SC increased 120% in the red soil and 86% in the yellow loamy soil^41^. The Cu and Zn concentrations in Chinese cabbage seedlings increased as a result of the application of composted pig manure. The treatment with the AP3-treated compost significantly increased the Zn concentration in the Chinese cabbage by 22% and 138%, compared with the control-treated compost and blank treatment, respectively. The average Cu concentration in the Chinese cabbage seedlings with treatments of five kinds of compost was 53% higher than that of blank treatment. Li *et al*. reported that CB treated compost improved Cu content of upground ryegrass by average of 27.2% compared with blank treatment while reduced Zn content by average of 6.4%^12^. Hua *et al*. reported that Cu content in fescue can be increased significantly by 123% and 208% when grown in red soil and yellow loamy soil, respectively, while ryegrass can be increased significantly by 88% and 86% with the addition of 80% SC. For Zn content, that in fescue can be increased significantly by 378% and 313% that grown with red soil and yellow loamy soil, respectively, while ryegrass can be increased significantly by 338% and 340% with the addition of 80% SC^41^. The reports indicated that the content of Zn and Cu in plant tissues might be influenced by the plant species, soil types, and compost addition in such pot trials. In our study, the AP amended PM compost can provide an efficient way for the available Zn enhancement in the typical Zn-deficient soil and for improvement of the Zn content in food crops since many Asian populations experience Zn deficiency^42^.

**Table 1:** Effect of compost addition on the growth of Chinese cabbage in pot experiment.

Treatments	SPAD	Dried biomass (g pot^−1^)	Zn concentration (mg kg^−1^)	Cu concentration (mg kg^−1^)
Blank	32.5 ± 2.2 d	0.85 ± 0.18 d	22.4 ± 1.5 d	3.8 ± 0.7 b
Control	42.2 ± 1.5 c	2.34 ± 0.15 c	43.6 ± 2.1 c	5.3 ± 0.5 a
AP1	45.6 ± 2.1 bc	2.86 ± 0.20 b	45.8 ± 1.8 bc	5.5 ± 0.9 a
AP2	48.5 ± 2.1 b	2.75 ± 0.16 b	48.2 ± 2.5 b	6.2 ± 0.5 a
AP3	52.6 ± 1.6 a	3.26 ± 0.19 a	53.4 ± 1.9 a	5.9 ± 0.8 a
CA	47.5 ± 1.8 b	2.55 ± 0.21 bc	44.5 ± 1.5 bc	6.1 ± 0.7 a

Note: Values indicate mean ± standard deviation based on the samples with four replications. Data in a column with the same letter mean there were no significant differences at *p* < 0.05.

## Conclusions

This study indicated that during composting, compared with the control and CA treatment, AP addition in composting matrix can improve TKN concentration and conserve N content by increasing ${{\rm{NH}}}_{4}^{+}$-N and ${{\rm{NO}}}_{3}^{-}$-N contents. The gaseous emissions of NH_3_ and N_2_O and the accumulation emission were inhibited with AP addition caused by the acid material input at the beginning of composting. As an important agricultural by-product, AP can be regarded as a suitable additive for pig manure aerobic composting with the aim of agricultural waste utilization and compost quality improvement, while the addition ratio should be monitored with the quality evaluation of pH, EC, N conservation and the economic benefit.

## Materials and Methods

### Raw materials

The fresh PM was collected from the Northwest A&F University campus’s ecological testing farm, Yangling, Shaanxi, China. WS was collected from the village around the campus (within 5 km) and crushed to 3–5 cm fragments before being mixed with the pig manure. Fresh apple pomace (AP) was provided by Tongda fruit plant (Xianyang City, Shaanxi Province) which is 50 km from the campus and citric acid (CA, Chemical pure) was purchased from Sinopharm Chemical Reagent Co., Ltd. The main characteristics of the raw materials are shown in Table 2.

**Table 2:** Initial properties of composting materials.

Parameters	Pig Manure	Wheat Straw	Apple Pomace
Moisture content (%)	71.22 ± 0.12	12.51 ± 0.02	7.82 ± 0.01
pH	8.42 ± 0.02	7.31 ± 0.02	3.94 ± 0.01
EC (mS cm^−1^)	3.56 ± 0.03	0.16 ± 0.01	0.25 ± 0.01
Particle size (mm)	ND	30–45	<0.30
TKN (g kg^−1^)	27.63 ± 0.41	3.94 ± 0.11	10.10 ± 0.13
TP(g kg^−1^)	17.12 ± 0.22	1.97 ± 0.03	0.82 ± 0.01
TK (g kg^−1^)	6.67 ± 0.05	11.72 ± 0.12	13.52 ± 0.08
OM (g kg^−1^)	535.3 ± 1.2	712.7 ± 2.2	852.9 ± 2.6
C/N	11.2	107.0	48.9
Zn (mg kg^−1^)	1123.6 ± 15.2	6.5 ± 0.2	15.2 ± 0.6
Cu (mg kg^−1^)	690.2 ± 7.4	9.5 ± 0.2	11.5 ± 0.5

Note: Values indicate mean ± standard deviation based on the samples with four replications.

### Composting procedure and sampling

About 63 kg of PM was mixed with 6 kg WS on wet weight ratio of 10.5: 1^17^ for each treatment, and then AP was supplemented 0%, 5%, 10% and 20% in dry weight basis, while the treatment of CA was conducted with citric acid adding rate of 1% in dry weight basis of the mixture and denoted as control, AP1, AP2, AP3, and CA, respectively. All treatments were performed in triplicate. The moisture of the mixtures in all treatments was kept around 65% after fully mixing. After that, about 60 kg mixture was put into a laboratory automatic-mixing composter (volume 120 L). Air was pumped from the bottom into the composter with a constant air flow of about 60 L min^−1^ during the thermophilic phase, and the frequency of ventilation was twice per day for 30 mins, once at about 9 a.m. and the other at about 3 p.m. Temperature was monitored every 8 h by a probe fixed in the middle of the composting mass and averaged. The room temperature was also recorded. Samples around 1 kg were collected at scheduled times (0, 6, 11, 16, 23, and 30 days) according to the change of temperature after the start of the experiment. Before sampling, all the piles were thoroughly turned. Sampling was performed in triplicate and each sample was divided into two portions. One portion was stored at 4 °C before analyses while the other was freeze-dried, crushed to pass through a 0.15 mm sieve and stored in desiccator. Another fresh sample portion was used for parameters including moisture, pH, EC, ${{\rm{NO}}}_{3}^{-}$-N, ${{\rm{NO}}}_{2}^{-}$-N, ${{\rm{NH}}}_{4}^{+}$-N, GI etc. determination. Gas samples were collected every day with a 1-LTedlar^®^ PLV gas sampling bag as described by Awasthi *et al*.^20^.

### Pot experiment

The pot experiment was conducted in the greenhouse of Northwest A & F University. The soil used in the pot experiment was a calcareous loam that collected from Yangmazhuang village, Yongshou County, Shaanxi Province, with the 0–20 cm depth sampling. The main characteristics of the soil are shown in Table 3. Air-dried soil and composts were ground to pass 2 mm nylon sieve before mixing. All the compost was added to soil with 5 wt.% application dosage as in our previous study^12^ except the blank treatment (only soil was added to the pot, without any fertilizer input), and the main characteristics of the matured compost are shown in Table 3. A plastic basin (the bottom diameter = 19 cm and the mouth diameter = 30 cm, height = 23 cm) filled with 2.0 kg soil/compost mixtures was incubated for 10 days with water content of around 80% field capacity, fifteen Chinese cabbage (*Brassica chinensis* L.) seeds were planted while thinned to 5 per pot at the growth stage of four leaves. Four replications were set for each treatment. During growth period, chlorophyll contents in the leaves were recorded by using a SPAD meter (Minolta, Japan-SPAD-502). After 35 days, the aboveground biomass of each pot was harvested. Plant samples were dried at 60 °C in an oven to constant weight for the dry biomass determination and stored in a dehydrator before Cu and Zn accumulation assessment.

**Table 3:** Initial properties of soil and matured compost.

Parameters	Soil	Control	AP1	AP2	AP3	CA
pH	8.40 ± 0.02	8.34 ± 0.02	8.27 ± 0.01	8.14 ± 0.02	7.71 ± 0.01	8.16 ± 0.01
OM (g kg^−1^)	16.1 ± 0.2	557.4 ± 1.6	548.8 ± 1.2	556.0 ± 2.4	558.4 ± 1.8	534.3 ± 1.4
TKN (g kg^−1^)	1.20 ± 0.06	24.87 ± 0.32	26.26 ± 0.22	28.60 ± 0.35	29.64 ± 0.16	26.19 ± 0.18
Olsen-P (g kg^−1^)	0.03 ± 0.00	2.10 ± 0.02	2.30 ± 0.04	2.23 ± 0.01	1.97 ± 0.03	2.28 ± 0.02
available K (g kg^−1^)	0.14 ± 0.01	13.82 ± 0.05	13.62 ± 0.06	13.84 ± 0.12	12.56 ± 0.05	13.33 ± 0.08
Total Cu(mg kg^−1^)	45.0 ± 0.6	513.3 ± 2.5	506.9 ± 1.8	487.4 ± 4.6	453.0 ± 3.8	493.3 ± 5.2
Total Zn(mg kg^−1^)	61.0 ± 1.2	821.6 ± 4.5	816.7 ± 8.4	752.6 ± 6.2	744.4 ± 5.6	802.1 ± 3.1
DTPA-Cu(mg kg^−1^)	0.5 ± 0.1	193.0 ± 2.6	165.3 ± 3.4	131.6 ± 1.8	134.1 ± 2.6	151.0 ± 1.6
DTPA-Zn(mg kg^−1^)	0.2 ± 0.0	210.3 ± 3.2	184.6 ± 2.5	152.9 ± 1.9	145.9 ± 3.5	167.6 ± 2.4

Note: Values indicate mean ± standard deviation based on the samples with four replications.

### Analytical methods

${{\rm{NH}}}_{4}^{+}$-N, ${{\rm{NO}}}_{3}^{-}$-N, ${{\rm{NO}}}_{2}^{-}$-N, moisture, EC, and pH were determined with fresh samples as methods used by Li *et al*.^4^ ${{\rm{NO}}}_{2}^{-}$-N content was measured by using a colorimeter at wavelength 538 nm^43^. Total Kjeldahl nitrogen (TKN) was monitored in air-dried samples followed by the Kjeldahl digestion method^4^. A mass balance was carried out before and after composting to calculate all results for each treatment. The N_2_O content was analyzed with gas chromatography (Agilent Technologies 6890 N Network GC system, China) in 12 h as described by Colon *et al*.^44^ Ammonia emission was measured by adsorbing the exhaust gas with H_3_BO_3_ before titrated with HCl^3^. Phytotoxicity of the compost was evaluated by calculating germination index (*GI*) as described by Li *et al*.^4^ with potherb mustard (*Ardisia squamulosa Presl*) as an indicator plant.

1 $$GI=\frac{({\rm{Seed}}\,{\rm{germination}}\,{\rm{rate}}\,{\rm{in}}\,{\rm{treatment}})\times ({\rm{root}}\,{\rm{length}}\,{\rm{in}}\,{\rm{treatment}})}{({\rm{Seed}}\,{\rm{germination}}\,{\rm{rate}}\,{\rm{in}}\,{\rm{control}})\times ({\rm{root}}\,{\rm{length}}\,{\rm{in}}\,{\rm{control}})}$$

The total contents of Cu and Zn in compost and in the plant samples were determined by an atomic absorption spectrophotometer (Hitachi Z-2000) after being digested by concentrated HNO_3_-H_2_O_2_. Reference samples (GBW-08501) were used for quality control meanwhile.

All analyses were repeated four times. Statistical analysis was performed by SPSS 18.0 software using one-way ANOVA at significance *P* < *0*.*05* level to separate treatment means for the mass data.
